# Characterizing piggyBat—a transposase for genetic modification of T cells

**DOI:** 10.1016/j.omtm.2022.03.012

**Published:** 2022-03-22

**Authors:** Gaurav Sutrave, Ning Xu, Tiffany C.Y. Tang, Alla Dolnikov, Brian Gloss, David J. Gottlieb, Kenneth P. Micklethwaite, Kavitha Gowrishankar

**Affiliations:** 1 Westmead Institute for Medical Research, Sydney, NSW, Australia; 2 Sydney Medical School, The University of Sydney, Sydney, NSW, Australia; 3 Children's Cancer Institute, University of New South Wales, Sydney, NSW, Australia; 4 School of Women's and Children's Health, UNSW Sydney, Sydney, NSW, Australia; 5 Blood Transplant and Cell Therapies Program, Department of Haematology, Westmead Hospital, Hawkesbury Road, Westmead, NSW 2145, Australia; 6 Blood Transplant and Cell Therapies Laboratory, NSW Health Pathology – ICPMR Westmead, Sydney, NSW, Australia

**Keywords:** chimeric antigen receptor, CAR, transposons, non-viral vector, piggybat, T cell immunotherapy, CD19

## Abstract

Chimeric antigen receptor (CAR) T cells targeting CD19 have demonstrated remarkable efficacy in the treatment of B cell malignancies. Current CAR T cell manufacturing protocols are complex and costly due to their reliance on viral vectors. Non-viral systems of genetic modification, such as with transposase and transposon systems, offer a potential streamlined alternative for CAR T cell manufacture and are currently being evaluated in clinical trials. In this study, we utilized the previously described transposase from the little brown bat, designated *piggyBat*, for production of CD19-specific CAR T cells. *PiggyBat* demonstrates efficient CAR transgene delivery, with a relatively low variability in integration copy number across a range of manufacturing conditions as well as a similar integration site profile to *super-piggyBac* transposon and viral vectors. *PiggyBat*-generated CAR T cells demonstrate CD19-specific cytotoxic efficacy *in vitro* and *in vivo.* These data demonstrate that alternative, naturally occurring DNA transposons can be efficiently re-tooled to be exploited in real-world applications.

## Graphical abstract

**Figure:**
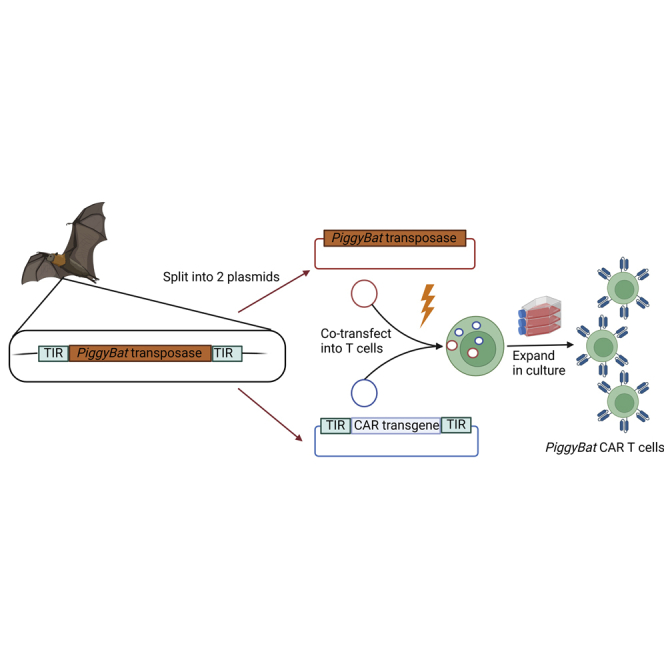

## Teaser

PiggyBat transposase can be utilized as a gene-modification tool for the efficient manufacture of chimeric antigen receptor T cells. It has a relatively low efficiency of transgene integration but demonstrates tighter control of vector copy number per cell, highlighting its potential for clinical applications.

## Introduction

Manufacture of chimeric antigen receptor (CAR) T cells is most commonly performed using lentiviral or γ-retroviral vectors. These viral vectors have an established record of efficiency and clinical safety but have several limitations, including high cost, constrained scalability with current technologies,^1^ and restricted transgene payloads of 10–14 kb,^2^ which is of relevance for more complex genetic modifications required to enhance CAR T cell efficacy (reviewed in Srivastava and Riddell^3^, ^4^) and safety.^5^, ^6^, ^7^

DNA transposons are naturally occurring genetic sequences consisting of a coding sequence for a transposase, flanked by repetitive sequences called terminal inverted repeats (TIRs). The transposase specifically binds to its associated TIRs and excises the intervening segment of DNA, pasting it into a distant part of the genome.

This system can be used for CAR T cell manufacture by separating the transposase coding sequence (onto either mRNA or a DNA plasmid) and inserting a CAR transgene between the TIRs on a separate plasmid. Introducing both these elements into T cells allows for the transposase to mediate stable chromosomal integration of the CAR transgene from the transposon plasmid.

Transposon and transposase systems offer several potential advantages over viral vectors for CAR T cell manufacture, including reduced cost and larger cargo capacity (up to 200 kb).^8^ These systems are also readily amenable to optimization, including development of high-efficiency transposase variants and more compact-sized transposons, allowing for more effective gene modification.^9^, ^10^, ^11^, ^12^, ^13^ Transposon and transposase systems display non-random genomic integration, although each different system has its own predilection for integration into specific genomic features that can potentially influence genotoxicity.^14^, ^15^, ^16^ Methods for the manufacture of CAR T cells have been described for transposon and transposase systems, including *Tol2*,^17^ *super-piggyBac*,^18^ and *Sleeping Beauty*,^19^ and the safety and efficacy of these manufacturing methods are being investigated in clinical trials.^20^, ^21^, ^22^, ^23^, ^24^

Naturally occurring DNA transposons are common, accounting for up to 25% of some amphibian genomes. While the majority of these transposons are no longer active, many can be re-engineered, potentially providing tools for manufacture of future gene therapies. The screening of mammalian genomes has led to the identification of an active transposase in the little brown bat, *Myotis lucifugus*.^25^^,^^26^ This “*piggyBat*” transposase has been shown to be capable of transposition in several organisms, including human cell lines.^27^

In this study, we investigated whether the *piggyBat* transposase could be used to generate CD19-specific CAR (CAR19) T cells. Using T cell electroporation for nuclear import of a CAR19 transgene followed by CAR-specific stimulation, we show that *piggyBat*-engineered T cells stably express a CAR19 with a relatively low number and narrow range of transgene integrations per cell and demonstrate CD19-specific activity *in vitro* and in murine xenograft models. These results suggest that *piggyBat* transposase could be used as an alternative gene-modification platform for *in vitro* evaluation of CAR design and after suitable safety testing for clinical application.

## Results

### *PiggyBat* transposase is capable of mediating stable integration of CD19-specific CAR construct into primary T cells

**Figure 1:**
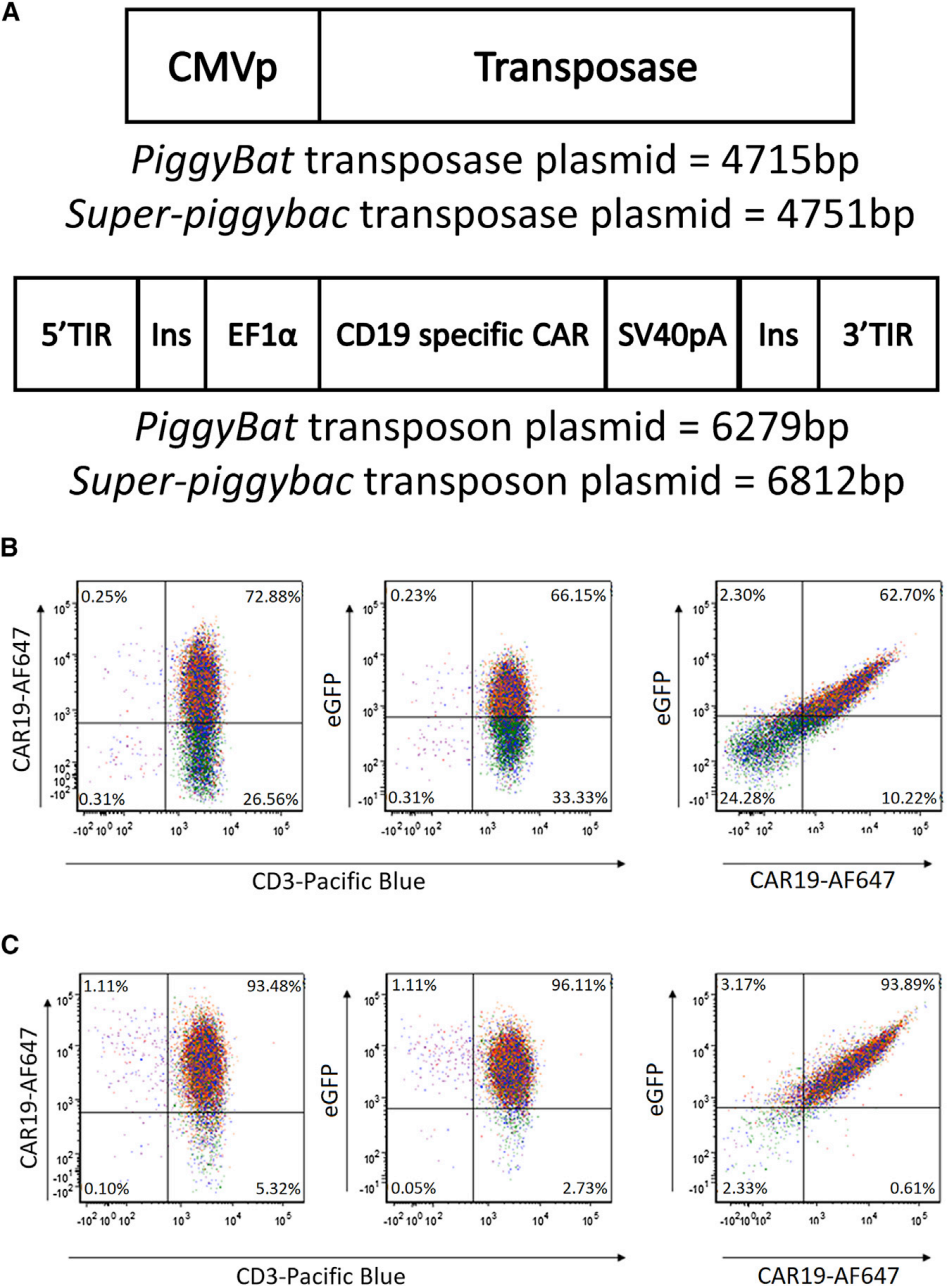
Generation of CD19-specific CAR T cells with piggyBat transposon and transposase system (A) Schematic of transposase (above) and transposon (below) plasmids used for PBat CAR19 and SPB CAR19 generation. (B) Representative flow plots of piggyBat-generated CD19-specific CAR T cells, co-expressing EGFP at the end of 2-week culture period (PBat CAR19h28TM41BBz2AGFP/PBat CAR19) are shown. (C) Representative flow plots of super-piggyBac-generated CD19-specific CAR T cells, co-expressing EGFP at the end of 2-week culture period (SPB CAR19h28TM41BBz2AGFP/SPB CAR19) are shown. 3′ TIR, 3ʹ terminal inverted repeat; 5′ TIR, 5ʹ terminal inverted repeat; CMVp, cytomegalovirus promoter; EF1α, human elongation factor-1α promoter; Ins, chicken β-globin insulator; SV40pA, simian vacuolating virus 40 poly-adenylation tail.

CAR T cells were produced from healthy peripheral blood mononuclear cells (PBMCs) using a *piggyBat* transposon and transposase plasmid pair with the transposon plasmid containing the *piggyBat*-specific TIR sequences,^27^ flanking a previously described second-generation CAR19 construct with a 4-1BB costimulatory domain^18^ and enhanced green fluorescent protein (EGFP) to enable CAR T cell detection (CAR19h28TM41BBz2AGFP). Production of these CAR T cells using *piggyBat* (PBat CAR19) was directly compared with the *super-piggyBac* transposon system (SPB CAR19) (Figure 1) using previously described protocols.^28^

**Figure 2:**
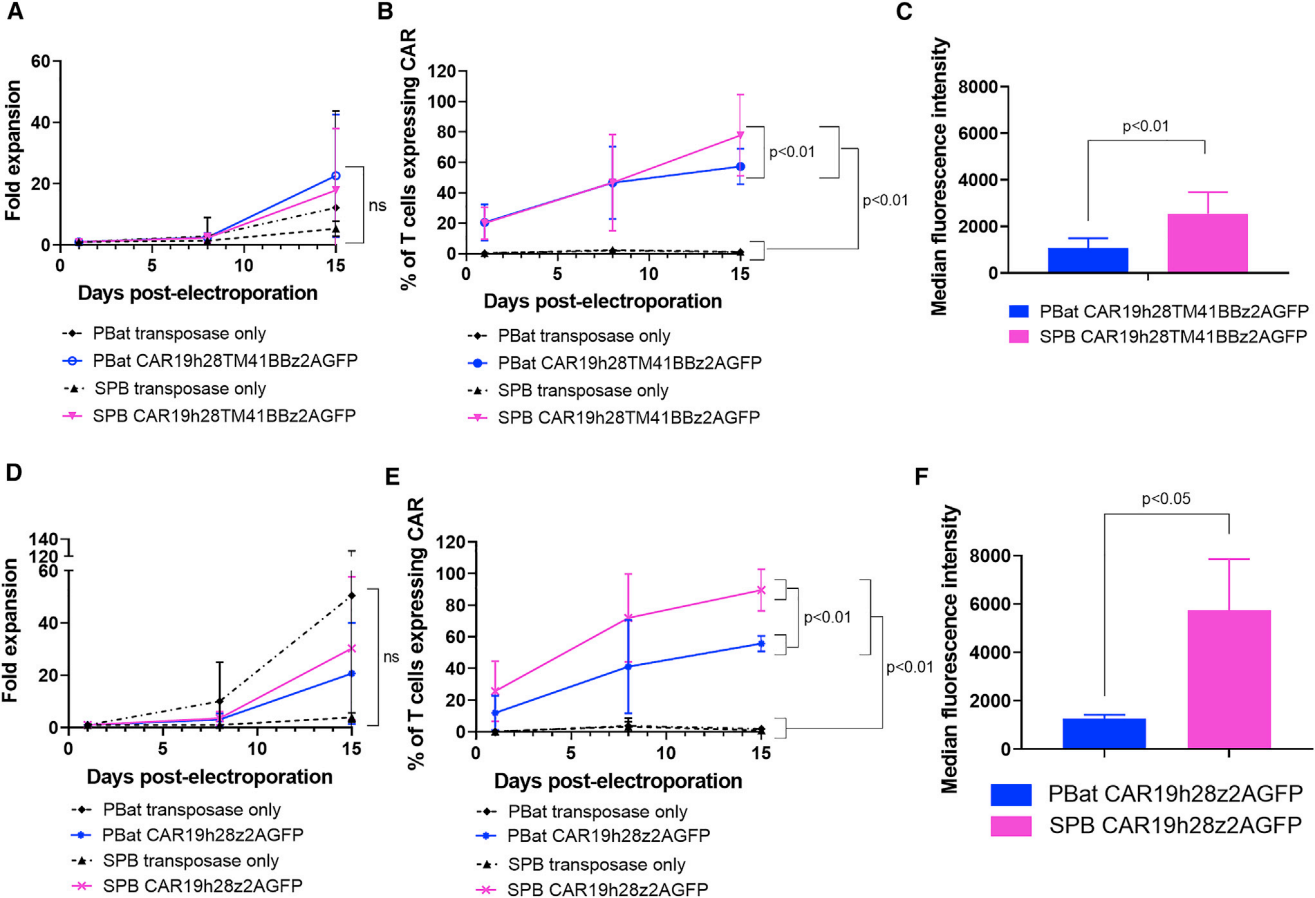
PBat-generated CD19-specific CAR T cell culture characteristics (n = 19 per condition) (A–C) Expansion (A), CAR expression (B), and MFI (C) of second-generation CD19-specific CAR T cells with 4-1BB containing co-stimulatory domain over a 2-week culture period following electroporation with piggyBat or super-piggyBac transposase and transposon (n = 19 per condition). (D–F) Expansion (D), CAR expression (E), and MFI (F) of different CD19-specific CAR T cell construct with CD28 co-stimulatory domain over a 2-week culture period (n = 3 per condition) are shown. Data are presented as mean ± standard deviation.

There was no difference in expansion between SPB CAR19 and PBat CAR19 at the end of 2 weeks culture (18-fold versus 23-fold, respectively; p = 0.902; Figure 2A). Level and intensity of CAR expression detected by flow cytometry with scFv-specific antibody correlated with EGFP expression on both PBat CAR19 and SPB CAR19 (Figures 1C and 1D). However, at the end of a 2-week culture, both percentage of CAR^pos^ T cells and median fluorescence intensity (MFI) of CAR expression (detected by EGFP) were lower with *piggyBat* compared with *super-piggyBac* (CAR expression 57.4% versus 77.8%, p < 0.01, and MFI 1,058 versus 2,542 arbitrary units, p < 0.01, respectively; Figures 2B and 2C). Lower CAR expression and MFI was also seen when CAR T cells were generated with a different second-generation CAR19 incorporating the CD28 costimulatory endodomain, with *piggyBat*-generated CAR T cells showing equivalent expansion (20- versus 30-fold; p = 0.999), reduced CAR expression (55.8% versus 89.5%; p = 0.001), and reduced MFI (1,686 versus 7,123 arbitrary units; p = 0.05) compared with *super-piggyBac*-generated CAR T cells after a 2-week culture (Figures 2D, 2E, and 2F, respectively). Production of CAR T cells with each transposase was specific to their corresponding transposon plasmid and TIR sequences, with no CAR T cells being produced when *piggyBat* transposase was combined with *super-piggyBac* transposon and vice versa (Figure S1).

**Figure 3:**
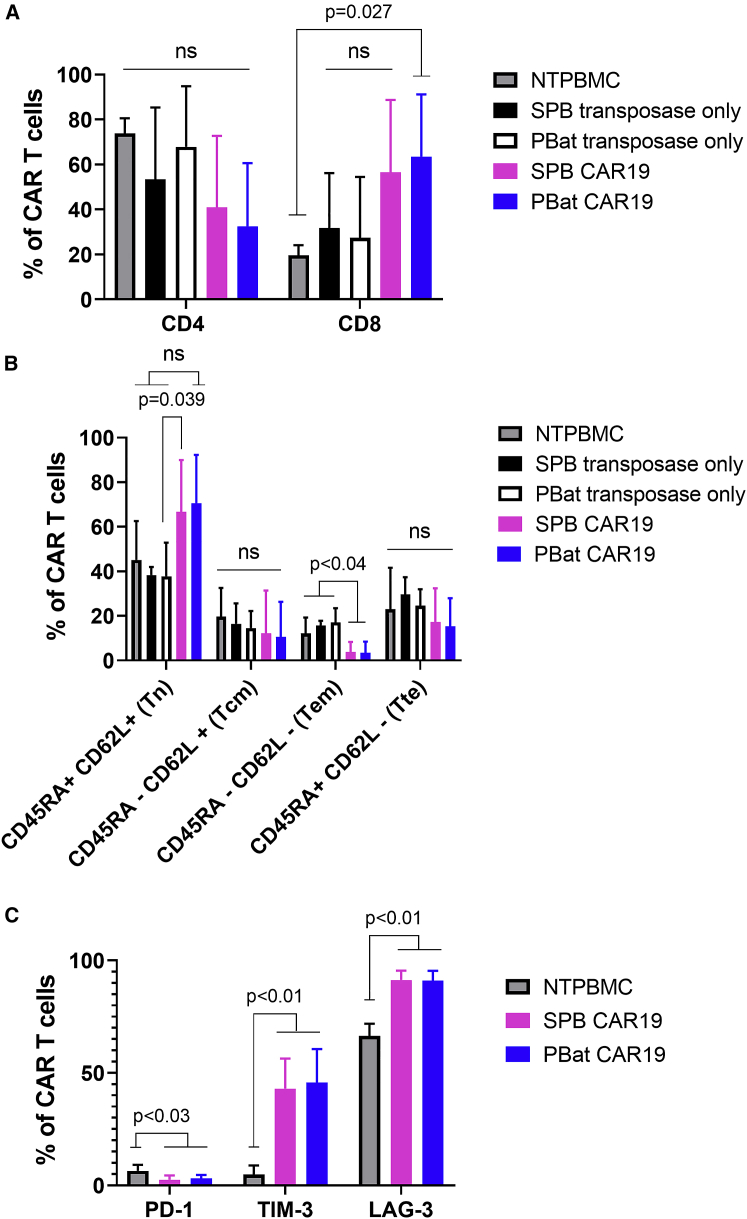
Phenotypic characteristics of PBat CAR19 T cells following 2 weeks culture Assessed by flow cytometry of CAR19 T cells. (A) CD4^pos^ and CD8^pos^ proportions in T cells cultured using piggyBat and super-piggyBac transposon and transposase systems are shown (n = 9 per condition). ns, not significant. (B) Memory phenotype of CAR T cells is shown (n = 9 per condition). Tcm, central memory; Tem, effector memory; Tn, naive; Tte, terminal effector. Comparison of Tem subset is as follows: SPB CAR19 versus SPB only: p = 0.007; SPB CAR19 versus PBat only: p = 0.001; SPB CAR19 versus NTPBMC: p = 0.038; PBat CAR19 versus SPB only: p = 0.005; PBat CAR19 versus PBat only: p = 0.001; PBat CAR19 versus NTPBMC: p = 0.029. (C) Expression of T cell co-inhibitory molecules on CAR T cells is shown (n = 6 per condition). No statistically significant different expression of PD-1 (3.0% versus 2.5% of CAR19 T cells), TIM-3 (45.7% versus 42.9%), or LAG-3 (91.0% versus 91.3%) between PBat CAR19 and SPB CAR19, respectively, is shown. Data are presented as mean + standard deviation.

Following 2 weeks culture, there was no difference in the phenotypic characteristics of PBat CAR19 and SPB CAR19, with both demonstrating a gradual increase in CD8^pos^ content over time (Figures S2A and S2B) to eventually be CD8^pos^ predominant at the end of culture (63.6% versus 56.5%; p = 0.963) compared with non-transfected PBMCs (NTPBMCs) or T cells electroporated with transposase plasmid only and cultured under identical conditions (Figure 3A). PBat CAR19 T cells show a favorable memory immunophenotype at the end of culture, consisting of a mean of 70.6% naive (Tn) and 10.6% central memory T (Tcm) cells, which was similar to that seen with SPB CAR19 (Figure 3B). While the relative proportion of Tn and Tcm CAR T cells increased between the start and end of culture, there was an accompanying reduction in effector memory T (Tem) cell content in both PBat CAR19 and SPB CAR19 cultures (Figures S2C and S2D) such that, by the end of culture, there was a significant reduction in Tem cells compared with NTPBMCs or T cells electroporated with transposase plasmid only.

There was no difference in expression of the T cell co-inhibitory molecules programmed cell death protein-1 (PD-1), T cell immunoglobulin and mucin domain containing-3 (TIM-3), and lymphocyte activation gene-3 (LAG-3) between PBat CAR19 and SPB CAR19. However, both TIM-3 and LAG-3 were overexpressed compared with NTPBMCs (Figure 3C). There was low expression of PD-1 in CAR19 T cells generated using both systems, which was lower than the level of expression on non-stimulated NTPBMCs.

### Levels of CAR expression can be controlled by altering amounts of *piggyBat* transposon and transposase

Further exploration of transduction conditions was undertaken to optimize production of PBat CAR19.

**Figure 4:**
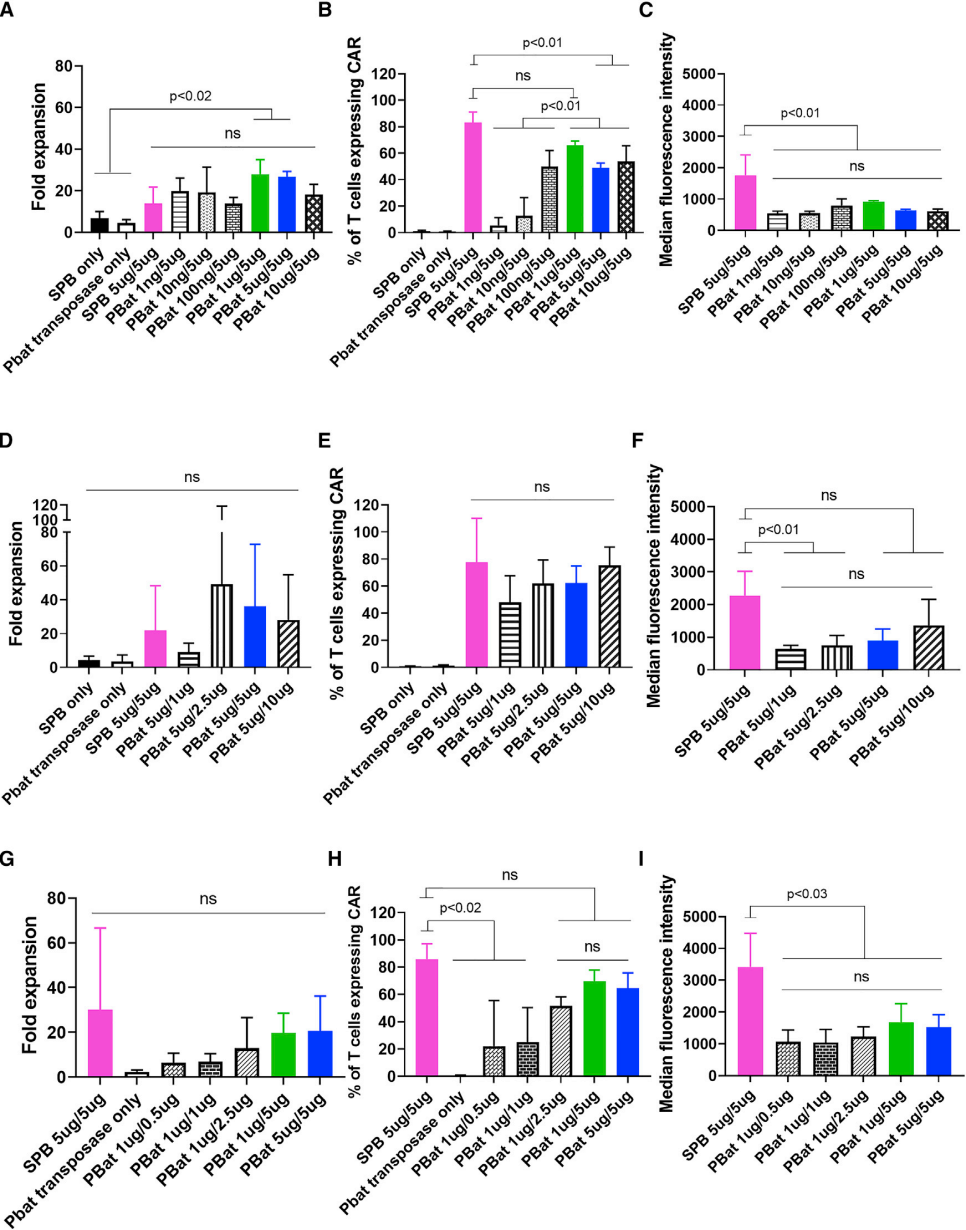
Optimization of PBat CAR19 transposon and transposase electroporation (n = 3 per condition) Mass of transposase plasmid utilized for CAR19 T cell manufacture indicated first with mass of transposase plasmid second. Increasing quantities of piggyBat transposase co-electroporated with fixed 5 μg of piggyBat transposon CAR19 plasmid results in equivalent expansion (A), with peak CAR19 expression (B) and MFI (C) achieved with 1 μg piggyBat transposase are shown. Increasing quantities of piggyBat transposon co-electroporated with a fixed 5 μg of piggyBat transposase plasmid results in equivalent expansion (D) with a trend toward increases in CAR19 expression (E) and MFI (F) with increasing transposon plasmid. Manufacture of PBat CAR19 with optimal 1 μg piggyBat transposase re-demonstrates equivalent expansion (G) with a trend toward transposon plasmid dose-dependent increase in CAR19 expression (H) and MFI (I). Data are presented as mean + standard deviation.

To evaluate *piggyBat* transposase overproduction inhibition as seen with other transposases,^29^ PBat CAR19 was generated with increasing amounts of *piggyBat* transposase plasmid while the amount of corresponding transposon plasmid was held constant. There was no consistent correlation between PBat CAR19 expansion over a 2-week culture and the quantity of *piggyBat* transposase plasmid electroporated (range 4- to 28-fold; Figure 4A). There was a gradual increase in end-of-culture percentage CAR19 expression with increasing quantities of *piggyBat* transposase (range 5.5% versus 66.0%; Figure 4B), with peak expression achieved by electroporation of 1 μg of *piggyBat* transposase with 5 μg of corresponding transposon plasmid, which achieved equivalent CAR expression as SPB CAR19 (66.0% versus 83.1%; p = 0.256). Further increases in *piggyBat* transposase resulted in a non-significant reduction in percentage CAR expression. The MFI of PBat CAR19 did not vary with amounts of *piggyBat* transposase electroporated and remained less than that of SPB CAR19 (range 544–905 arbitrary units versus 1,755 arbitrary units; p < 0.02; Figure 4C).

The transposition process is mediated through an enzymatic reaction catalyzed by the transposase enzyme acting on the transposon. To test the hypothesis that increasing the transposon substrate will result in increased transposition and CAR integration into the T cell genome, increasing amounts of *piggyBat* transposon plasmid were electroporated with a fixed 5 μg of *piggyBat* transposase plasmid. PBat CAR19 expansion remained equivalent to SPB CAR19 over a 2-week culture period, regardless of the quantity of transposon plasmid utilized (range 9- to 49-fold versus 22-fold; Figure 4D). There was no significant increase in CAR expression (range 48.0%–75.4%; Figure 4E) or MFI (range 642–1,362 arbitrary units; Figure 4F) at the end of a 2-week culture with increasing quantities of transposon plasmid.

This same trend was confirmed when PBat CAR19s were generated with increasing quantities of *piggyBat* transposon plasmid with 1 μg of *piggyBat* transposase plasmid (Figures 4G–4I). A combination of 1 μg *piggyBat* transposase plasmid and 5 μg of *piggyBat* transposon plasmid achieved peak CAR19 expression of 69.5% and MFI of 1,672 arbitrary units at the end of culture.

### *PiggyBat* transposase demonstrates titratable genomic integration with a similar pattern of integration as *super-piggyBac* and viral vectors

**Figure 5:**
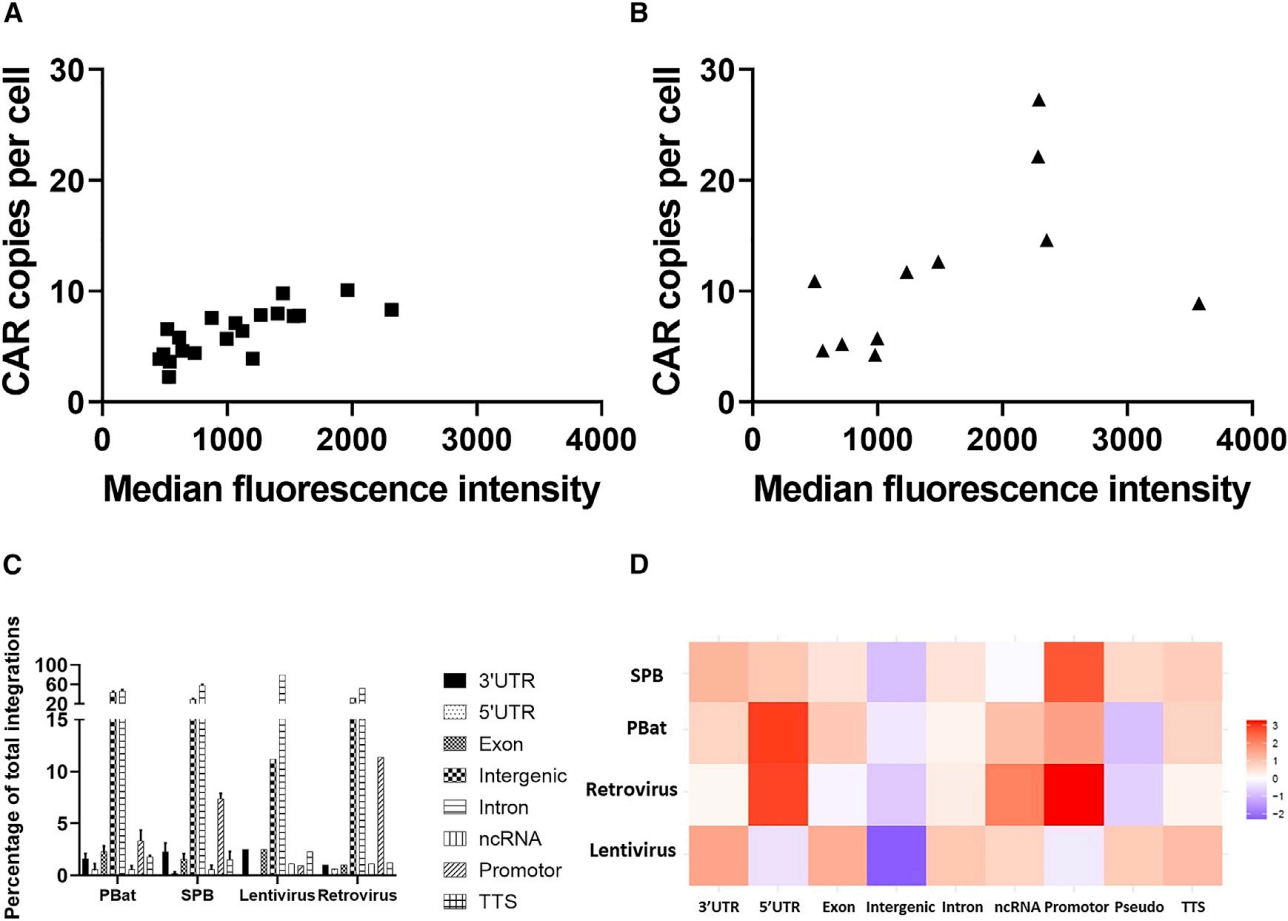
PBat CAR19 demonstrates titratable integration copy number with similar pattern of integration to SPB and viral vectors (A and B) CAR19 integration copy number as a function of MFI for (A) PBat CAR19 and (B) SPB CAR19. (C) Total number and distribution of unique integration sites of CAR19 T cells generated using *piggyBat* and *super-piggyBac* systems are shown (n = 3 per condition). (D) Heatmap comparing relative observed integrations into different classes of chromosomal loci to a random integration pattern for *piggyBat* (PBat), *super-piggyBac* (SPB), and published lentiviral and retroviral integration sites is shown.^30^ Each row represents a different gene-modification method, and each column represents different integration locus. Data are presented as mean + standard deviation. 3′ UTR, 3ʹ untranslated region; 5′ UTR, 5ʹ untranslated region; Inter, intergenic; ncRNA, non-coding RNA; prom, promoter; pseudo, pseudogene; TTS, transcription termination site.

To determine whether the reduction in CAR MFI with down-titration of PBat CAR19 transposon correlated with lower transposition activity, CAR19 transgene integration copy number was quantified by droplet digital PCR on genomic DNA extracted from CAR19 cultures following a 2-week culture period. In both PBat CAR19 and SPB CAR19 cultures, higher MFI was associated with an increase in mean CAR integration copy number per cell. PBat CAR19 displayed reduced mean CAR integration copy number per cell for any given MFI compared with SPB CAR19 (range 2.3–10.1 versus 4.3–27.3; Figures 5A and 5B) but showed a more consistent number of integrations with less donor-to-donor variability for a given amount of transposon (Figure S3A). The mean CAR integration copy number also appeared to increase with increasing quantities of transfected transposon plasmid (Figure S3B), in spite of equivalent transfection efficiency (Figure S4).

Integration site analysis was conducted by targeted next-generation sequencing of amplicons spanning the junction of *piggyBat* and *super-piggyBac* TIRs and adjacent genomic DNA from CAR T cells following 2 weeks culture. Identified integration sites were compared with those described for lenti- and retroviral vectors.^30^ PBat CAR19 demonstrated similar total unique integration sites per culture compared with SPB CAR19 (495 versus 653 total unique integration sites; p = 0.575). PBat CAR19 and SPB CAR19 demonstrate a similar pattern of integration, with the majority of insertions being in intergenic and intronic loci (Figure 5C). PBat CAR19 demonstrates a non-random pattern of integration, with a slight preference for integration within exons and 3′ untranslated regions relative to retrovirus (Figure 5D). When compared with lentivirus, *piggyBat* demonstrates a relative preference for integration 5′ untranslated regions and promoter and transcription start sites (Figure 5D). Conventional karyotyping conducted in PBat CAR19 and SPB CAR19 from two donors did not show any evidence of detectable structural chromosomal abnormalities with either transposon and transposase system (Figure S5).

### *PiggyBat*-generated CD19-specific CAR T cells demonstrate CD19-specific cytotoxicity *in vitro* and *in vivo*

**Figure 6:**
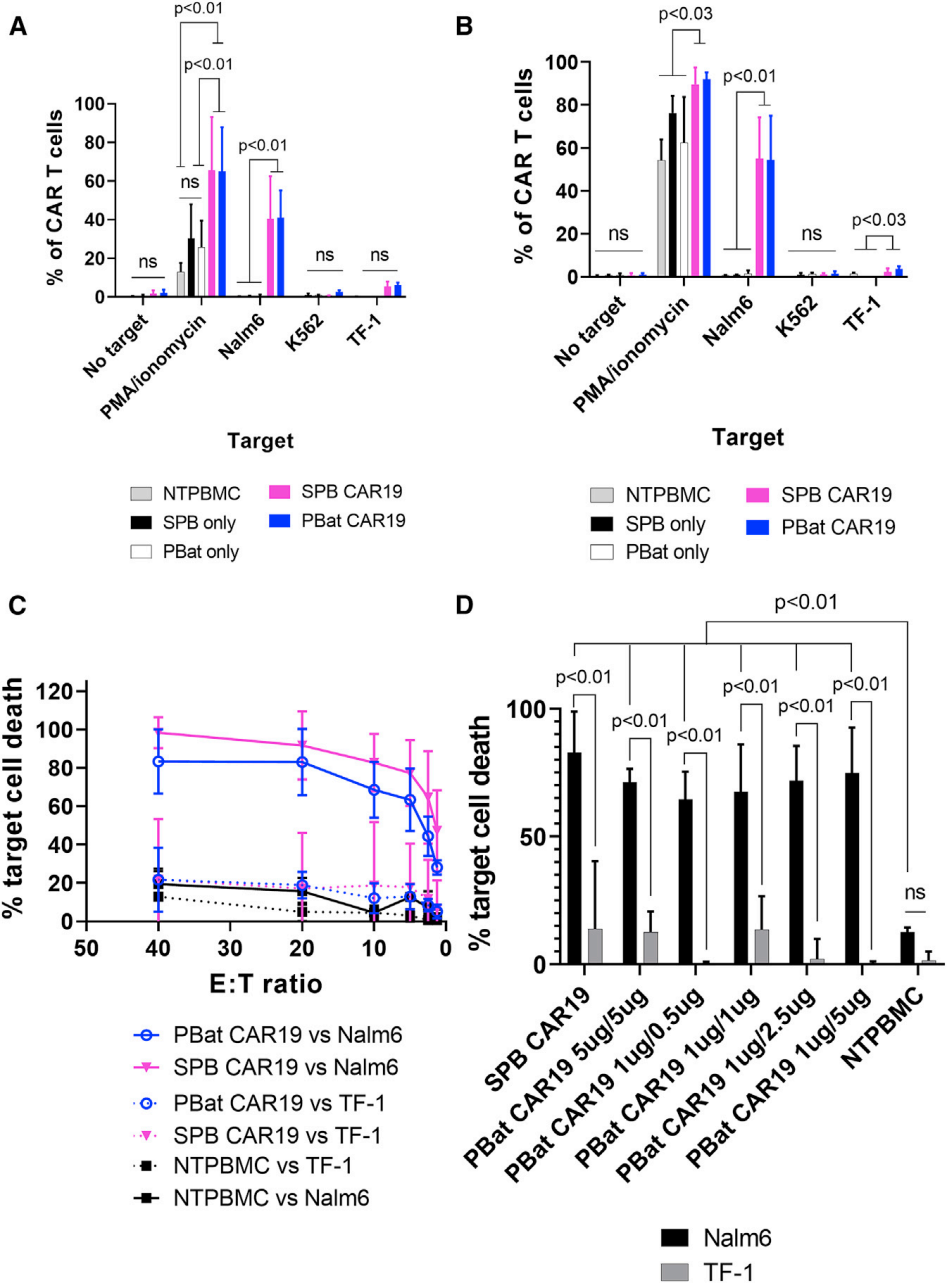
PBat CAR19s have CD19-specific cytotoxic activity (A and B) Intracellular cytokine production of (A) interferon γ and (B) tumor necrosis factor α as assessed by flow cytometry in PBat and SPB CAR19 following 1 h co-culture with different CD19^pos^ (Nalm6) and CD19^neg^ (K562 and TF-1) cell lines. PMA/ionomycin and no target serve as positive and negative controls, respectively (n = 5 per condition). (C) Calcein release assay demonstrating that SPB and PBat CAR19 T cells have CD19-specific, dose-dependent cytolytic activity following 4 h co-culture with target cell lines (CD19^pos^ Nalm6 and CD19^neg^ TF-1; n = 3 per condition). (D) PBat CAR19 manufactured with varying transposon concentrations demonstrates equivalent *in vitro* cytotoxicity to SPB CAR19 at an effector-to-target ratio of 10:1 (n = 3 per condition). Data are presented at mean ± standard deviation.

PBat CAR19 demonstrated specific T cell activation upon exposure to CD19-expressing cell lines. There was production of interferon γ and tumor necrosis factor α following co-culture with the CD19^pos^ Nalm6 cell line, but not CD19^neg^ cell lines (K562 and TF-1). Negative-control NTPBMCs and T cells cultured for 2 weeks following transfection with transposase alone showed activation with phorbol 12-myristate 13-acetate (PMA) and ionomycin, but not cell lines, confirming that cytokine production was CAR mediated (Figures 6A and 6B).

Cytotoxic capability of PBat CAR19 against CD19^pos^ cells was assessed by calcein-AM assay. PBat CAR19 demonstrated specific cytotoxicity of CD19^pos^ Nalm6 targets with minimal lysis of CD19^neg^ TF-1 targets similar to negative-control NTPBMC effector cells (Figure 6C).

We next examined whether the differences in CAR19 transfection conditions with *piggyBat* affected CAR T cell function. Cytotoxic activity of PBat CAR19 manufactured by electroporation with varying *piggyBat* transposon and transposase combinations was assessed at a fixed effector-to-target ratio of 10:1 (Figure 6D). This revealed that PBat CAR19 retained CD19 specificity with equivalent lysis of Nalm6 target cells, regardless of the percentage CAR expression or MFI across the range of transfected transposon masses tested.

**Figure 7:**
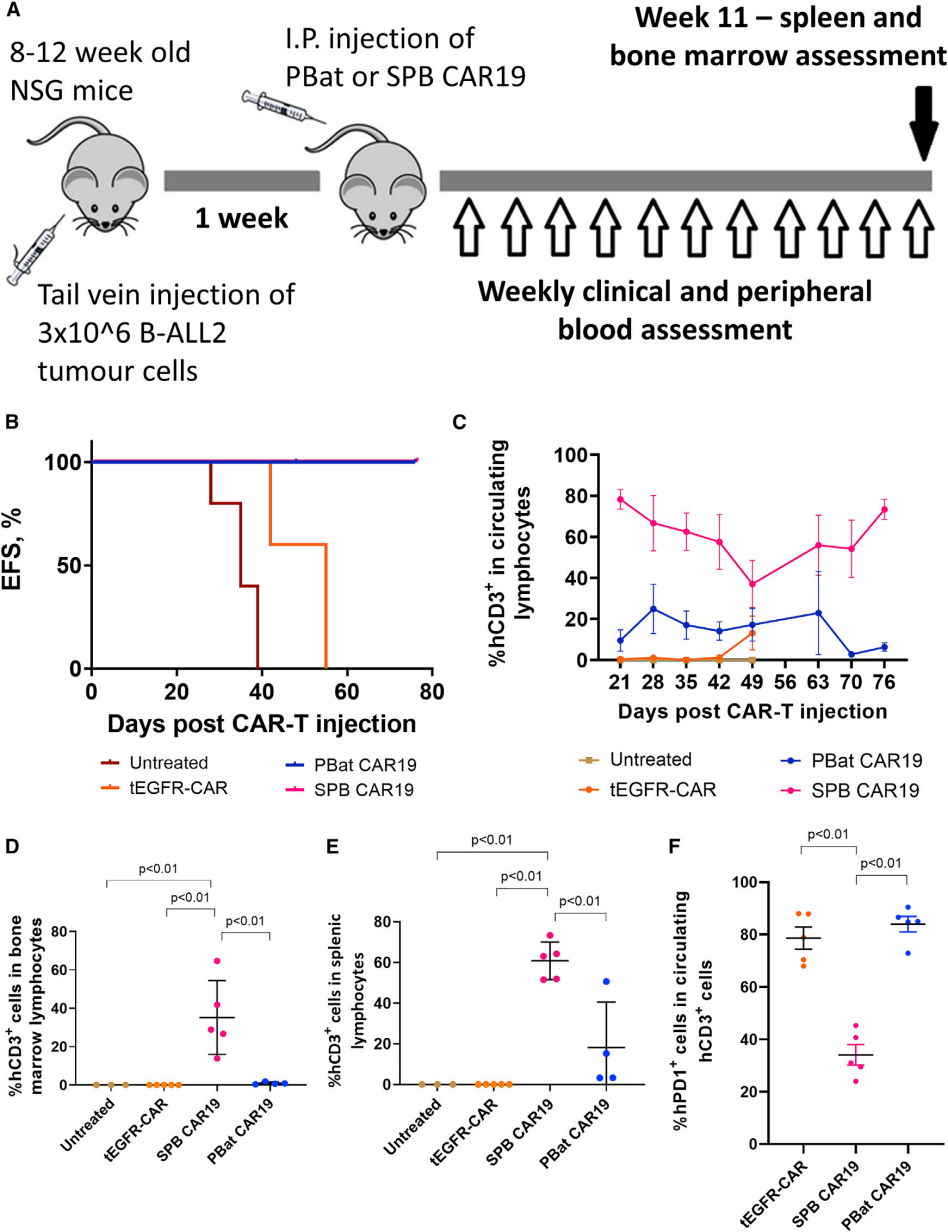
PBat CAR19s demonstrate *in vivo* efficacy in a murine PDX model (A) Schematic of PDX B-ALL murine model for assessment of *in vivo* efficacy of PBat CAR19. (B) Kaplan-Meier curve demonstrates prolonged leukemia-free survival with PBat CAR19 and SPB CAR19 compared with untreated and control (tEGFR-CAR)-treated mice (PBat CAR19 and SPB CAR19 curves overlap). (C–E) Proportion of human CD3^pos^ lymphocytes detectable in peripheral blood over time (C) and in bone marrow (D) and spleen (E) at end of 77 days or death in treated mice is shown. (F) Increased expression of human PD-1 on circulating tEGFR-CAR and PBat CAR19 relative to SPB CAR19 4 weeks following CAR T cell infusion is shown. Data are presented as mean ± standard deviation.

To assess the *in vivo* efficacy of PBat CAR19, PBMCs from a single healthy donor were used to manufacture PBat CAR19, SPB CAR19, and a control, second-generation CAR T cell expressing an extracellular truncated epidermal growth factor combined with a CD28 transmembrane and intracellular domain with CD3ζ (tEGFR-CAR) (Figure S6). A total of 0.33 × 10^6^ CAR-positive T cells were then infused in a chemoresistant B cell acute lymphoblastic leukemia (B-ALL) patient-derived xenograft (PDX) mouse model (Figure 7A). Both untreated mice and tEGFR-CAR-treated mice rapidly experienced a leukemia event (defined as >20% total nucleated cells in peripheral blood expressing human CD19) at a median of 35 and 55 days, respectively, while median event-free survival was not reached in mice treated with either SPB CAR19 or PBat CAR19 (Figure 7B; p < 0.01). Notably, PBat-CAR19-treated mice demonstrated a later (28 days versus 21 days) and lower peak T cell expansion compared with those treated with SPB CAR19 (24.89% versus 78.31% of total circulating lymphocytes; p = 0.022; Figures 5 and 7C). PBat CAR19 T cells also displayed higher PD-1 expression 4 weeks after infusion (83.96% versus 34.1% of circulating human T cells; p < 0.01; Figure 7F) and were found in reduced quantities in the bone marrow (0.88% versus 35.18% of bone marrow lymphocytes; p = 0.002; Figure 7D) and spleen (18.08% versus 60.8% of splenic lymphocytes; p = 0.001; Figure 7E) 11 weeks after infusion.

**Figure 8:**
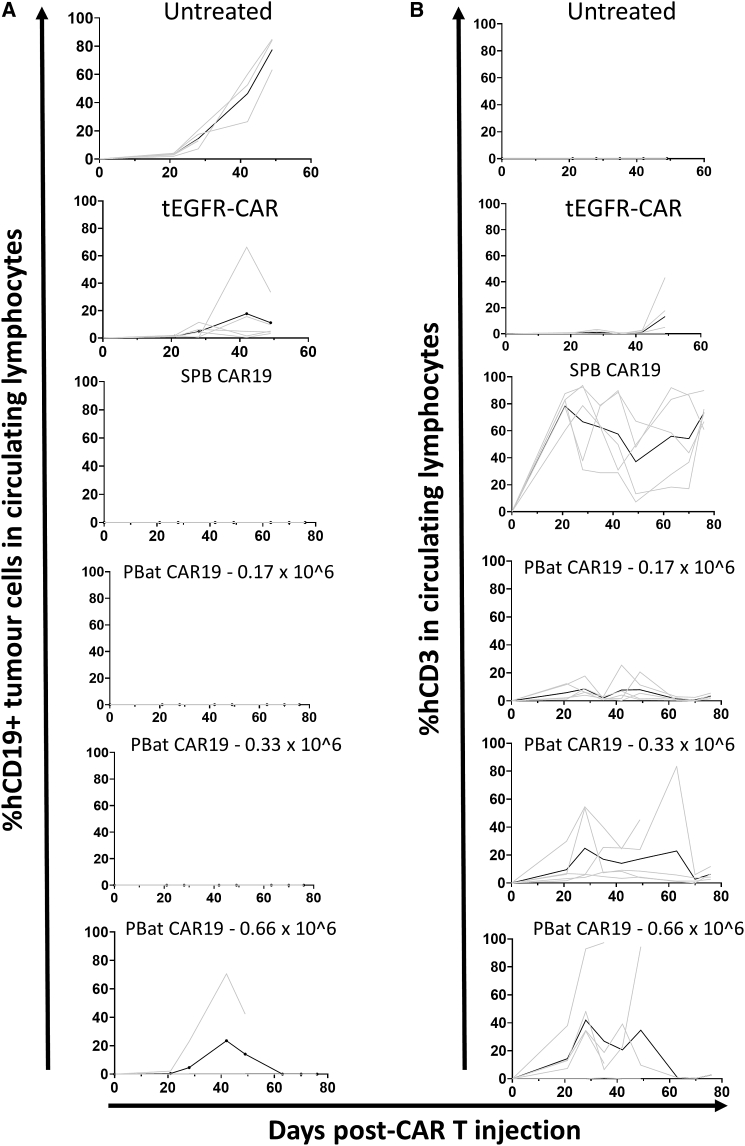
Suppression of CD19^pos^ tumor growth can be achieved with low-dose PBat CAR19 with equivalent efficacy as SPB CAR19 Circulating CD19^pos^ tumor cells (A) with corresponding detectable human CD3^pos^ T cells (B) in peripheral blood of treated mice over time. Data from each individual are in gray with mean for group in black. PiggyBat transposase can be utilized as a gene modification tool for the efficient manufacture of CAR T cells. It has a relatively low efficiency of transgene integration but demonstrates tighter control of vector copy number per cell, highlighting its potential for clinical applications.

Given the reduced numbers of PBat CAR19 T cells detected in leukemia-free mice after 11 weeks, we next sought to determine whether total CAR T cell dose would influence *in vivo* activity and CAR T cell persistence. Using the same murine model, five mice per group were administered either 0.17 × 10^6^ (CAR low), 0.33 × 10^6^ (CAR medium), or 0.66 × 10^6^ (CAR high) PBat CAR19 1 week following engraftment of B-ALL PDX cell line and compared with untreated mice. Control mice were administered 0.33 × 10^6^ tEGFR-CAR T cells and 0.33 × 10^6^ SPB CAR19. Event-free survival was similar between all PBat CAR19 doses, with only a single mouse in the CAR-high group experiencing a leukemia event (Figure 8A). Mice in the CAR-high PBat CAR19 group demonstrated a trend towards greater T cell proliferation in peripheral blood compared with the CAR-medium or CAR-low groups, although expansion was still less than that seen in the mice administered SPB CAR19 (Figure 8B).

While the CAR-high PBat CAR19 group mice displayed good leukemia control, three out of four mice in this group died of graft versus host disease without a leukemia event within 11 weeks of CAR T infusion (Figure S7). The final surviving mouse in this group had no evidence of leukemia and did not demonstrate any difference in T cells persistence in bone marrow or spleen at 11 weeks compared with the mice receiving the CAR-low or CAR-medium PBat CAR19 doses. Taken together, this suggests that escalating PBat CAR19 doses above a given threshold does not improve leukemia-free survival and may lead to greater toxicity mediated through mismatched T cell receptor (TCR)-human leukocyte antigen (HLA) interactions.

## Discussion

Transposons offer an alternative for genetic modification of cells and may overcome some of the limitations of viral vectors for CAR T cell manufacture. Transposon systems offer large transgene capacity and the ability to directly introduce new genetic modifications into cells without production of high-titer viral vectors, making them attractive tools for the rapid evaluation of novel gene-modified cellular therapeutics *in vitro* and in murine models. In addition, being plasmid based, the ability to directly modify and rapidly produce transposons coding for new constructs make them a facile laboratory tool for pre-clinical assessment of new CARs and cellular circuits. While transposon sequences are widespread in nature, few have been assessed for their utility in CAR T cell production and only the *Sleeping Beauty* and *super-piggyBac* systems have been assessed in clinical trials. The different characteristics of varying transposon systems in terms of their efficiency and patterns of genomic integration as well as cross-reactivity with endogenous human transposases could be leveraged to develop manufacturing protocols to generate safer CAR T cell products.

This current study demonstrates the manufacture of CAR19 T cells using the *piggyBat* transposon. *PiggyBat* CAR T cells demonstrate antigen-specific function *in vitro* and *in vivo*, with similar integration site distribution as *super-piggyBac* and viral vectors. Integrant copy number could be easily titrated by varying amount of transposon combined with an optimized *piggyBat* transposase concentration. The correlation between transposon concentration and integrant copy number was highly consistent across a wide range of transfected vector components, with low inter-donor variability. Similar to *super-piggyBac* and unlike mariner class transposases, such as *Sleeping Beauty*,^31^, ^32^, ^33^ the *piggyBat* transposase did not demonstrate overproduction inhibition across the range of transposase and transposon ratios tested.

The *piggyBat* system was optimized for the generation of CAR19 T cells over a 2-week culture period. This simple CAR-T-cell-manufacturing platform has the potential to allow for decentralized, small-scale CAR T cell production for use in clinical trials to take advantage of the short turnaround from leukapheresis to infusion,^34^ thereby reducing the potential for delays in treatment.

However, the development of T cell lymphoma in two patients receiving *super-piggyBac*-generated CAR19 T cells emphasizes the need for caution in translating new transposon systems into clinical use, particularly those with a similar pattern of insertion to *super-piggyBac*, as seen here.^35^ While the cause of malignant transformation of *super-piggyBac*-generated CAR T cells has not been unequivocally resolved, high transgene copy number per cell may have played a role in at least one of the two malignancies. The consistent and lower integrant copy numbers per cell across a broad range of transposon concentrations with the *piggyBat* system could attenuate this as a risk within the same culture system. Current regulatory guidelines for CAR T cells manufactured by viral vectors dictate an integrant copy number of less than 5 per cell, which could potentially be achieved by down-titration of transfected transposon plasmid. Further modifications, including the use of smaller transposon cassettes and transient transposase expression through mRNA transfection, have also been applied to minimize the risks of potential insertional mutagenesis.^36^ The availability of these approaches highlights the need for further optimization of the *piggyBat* system to satisfy regulatory requirements preceding clinical translation.

While these results suggest that *piggyBat* may offer features that make generation of genetically modified cells safer than that with the analogous *super-piggyBac* system, we note that, other than the *piggyBat*-specific inverted repeat sequences, the plasmids and manufacturing process used are the same as those used in the study in which malignancies occurred. There is also emerging recognition of a family of active *piggyBac* transposases in humans, with conflicting evidence regarding the ability of these transposases to recognize *piggyBac* family inverted repeat sequences.^37^^,^^38^ This raises the possibility of ongoing transgene remobilization and a heightened risk of insertional oncogenesis. Some of these studies suggest that *piggyBat* does not share cross-reactivity with endogenous human *piggyBac* family transposases, but the potential for mutagenesis related to all components of the *piggyBat* system will need to be thoroughly excluded and extensive pre-clinical safety testing would be required before clinical use of *piggyBat*-generated CAR T cells could be contemplated.

This study identifies a novel open-source transposase with several desirable gene-modification characteristics that can be utilized to manufacture CAR19 T cells while retaining *in vitro* and *in vivo* cytotoxic activity. The safety and applicability to other antigen targets will need to be confirmed preceding more widespread application of the *piggyBat* transposase for the manufacture of CAR T cell products. These results highlight the interesting potential for other as-yet-unidentified DNA transposons to be re-tooled for real-world applications.

## Materials and methods

### Plasmids

The *super-piggyBac* transposase (pVAX1-*super-piggyBac*) and transposon plasmids (pVAX1-PB) were as previously described.^18^ The *super-piggyBac* transposase (System Biosciences, Palo Alto, CA) is an engineered, hyperactive variant of the originally described *piggyBac* transposase.^39^ The *piggyBat* transposase sequence^27^ was synthesized by GenScript (Piscataway, NJ) and cloned into the multiple cloning site of the pVAX1 plasmid (Life Technologies, Carlsbad, CA, Catalog V260-20) using the restriction enzymes BamHI and XhoI and T4 DNA ligase from New England Biolabs (Ipswich, MA).

A *piggyBat* transposon expression vector (pVAX1-PBat) was created based on the previously described 5′- and 3′-TIRs sequences.^27^ The TIRs surrounded a 5′-and-3′ chicken β-globin insulator (cHS4),^40^ the mammalian elongation factor-1α promoter (UniProt: P68104), a multicloning site, and the simian virus 40 polyadenylation sequence. The expression cassette was directly synthesized (Genscript) and subcloned into the pVAX1 plasmid between the restriction enzyme sites AscI and XcmI.

### CD19-specific CAR constructs

Two second-generation CAR19 T cell constructs, designated CAR19h28TM41BBζ and CAR19h28ζ, were as previously described.^18^ Each CAR construct and an EGFP marker (UniProt: C5MKY7) were synthesized (Genscript) and cloned into the pVAX1-PBat transposon plasmid multicloning site through restriction enzyme digestion and ligation with T4 DNA ligase (New England Biolabs).

Cloning was confirmed by Sanger sequencing (Australian Genome Research Facility, North Melbourne, Australia).

### Cell lines

The CD19-positive Nalm6 (Deutsche Sammlung von Mikroorganismen und Zellkulturen [DSMZ] ACC-128) and CD19-negative TF-1 (ATCC CRL-2003) cell lines were kindly provided by Dr. Linda Bendall (University of Sydney, Australia). Nalm6 and TF-1 were both cultured in complete RPMI (cRPMI) consisting of RPMI-1640 (Lonza, Basel, Switzerland) with 10% heat-inactivated fetal bovine serum (FBS) (Serana, Bunbury, Australia), 2 mM L-glutamine (Sigma-Aldrich, St. Louis, MO), 100 units/mL penicillin G (Thermo Fisher Scientific, Waltham, MA), and 100μg/mL streptomycin (Thermo Fisher Scientific).

### CAR T cell generation

Ethics approval was obtained from the Sydney West Local Health District Research Ethics Committee for the collection of whole blood from healthy donors who had provided informed consent in accordance with the Declaration of Helsinki. PBMCs were isolated from whole blood using Ficoll density gradient centrifugation (GE Health Care, Chicago, IL) and cryopreserved in freezing media containing 70% phosphate buffered saline (PBS) (Lonza), 20% FBS (Serana), and 10% dimethylsulfoxide (DMSO) (Sigma-Aldrich). *Super-piggyBac* and *piggyBat* CAR19 T cells were generated from each donor as previously described.^18^ In brief, PBMCs were thawed and rested in culture medium consisting of AIM-V (Life Technologies) and 10% FBS (AIM V + FBS) for 24 h. Rested PBMCs were then electroporated using the Neon electroporation system (Life Technologies) with either *super-piggyBac* or *piggyBat* transposase and transposon plasmid using settings of single pulse, 20 ms, and 2400 V. One day following electroporation, and at weekly intervals thereafter, cells were enumerated by trypan blue (Sigma-Aldrich) exclusion, analyzed by flow cytometry, and re-cultured in AIM V + FBS with irradiated autologous PBMCs added for stimulation. Culture medium was supplemented with 200 international units/mL interleukin-15 (IL-15) (Miltenyi Biotec, Bergisch Gladbach, Germany) every 2 to 3 days, with media exchanges performed as needed. Cells were harvested 15 days post-electroporation and cryopreserved in freezing media until required for functional analysis.

### Phenotypic analysis

*Super-piggyBac* and *piggyBat* CAR19 cultures were phenotyped at weekly intervals from 24-h post-electroporation for 2 weeks for CAR and EGFP expression and T cell memory immunophenotype. The expression of T cell co-inhibitory molecules was assessed only at the end of culture. The following fluorochrome-conjugated anti-human monoclonal antibodies were used: CD3-PE/Cy7, CD4-BUV395, CD8-Pacific Blue, CD45RA-BV510, CD62L-APC, PD1-BV711, TIM3-APC/Cy7, and LAG3-PE (all from BD Biosciences), with EGFP being used as a surrogate for CAR19 expression. A 1:1 correlation between EGFP and CAR19 expression was confirmed through separate flow cytometric analysis by staining with CD3-Pacific Blue and CAR19scFv-specific monoclonal antibody^41^ conjugated to Alexa Fluor 647 (kindly provided by Prof. Laurence Cooper, MD Anderson Cancer Center, Houston, TX).

A total of 2 × 10^5^ cells from each culture condition were taken and washed and resuspended in PBA (PBS 1% [Sigma-Aldrich] and 0.05% sodium azide [Amresco, Solon, OH]) and stained with antibodies for 30 min at 4°C in the dark. Following staining, cells were again washed and resuspended in PBA. A minimum of 3 × 10^5^ total events were acquired using the LSR Fortessa (BD Biosciences) flow cytometer. Forward and side-scatter properties were used to discriminate between live and dead cells. Fluorescence minus one (FMO) controls were used to set gate positions as analyzed on FACSDiva (BD Biosciences) at time of data acquisition. Where required, more detailed analysis and graphic representation was conducted using FCS Express v.4 Research Edition (De Novo Software, Los Angeles, CA). MFI was calculated on gated CAR^pos^ cell populations and described in arbitrary units.

### Intracellular cytokine flow

Intracellular production of interferon γ and tumor necrosis factor α with CAR19 T cells or control non-transfected PBMCs following a 1-h co-culture with CD19-positive (Nalm6) and CD19-negative (TF-1) cell lines or control stimuli was performed as previously described.^28^

Antibodies used for detection were CD3-BV711, CD4-BUV395, CD8-Pacific Blue, interferon-γ-PE, and tumor necrosis factor-α-PE/Cy7 (all from BD Biosciences).

### CD19 CAR T cell cytotoxicity

Cytotoxicity was assessed by calcein release assay as previously described.^42^ Effectors (either CAR19 T cells or non-transfected PBMCs) were co-cultured for 1 h with K562 cells at a 1:1 ratio in AIM-V + FBS to adsorb NK cell activity. Target cells (either Nalm6 or TF-1) were labeled with 25 μM Calcein-AM (Sigma-Aldrich) for 30 min, following which target cells were washed and resuspended in cRPMI without phenol red (Lonza). A total of 1.5 × 10^4^ targets were then incubated in triplicate with effectors at increasing effector-to-target ratios ranging from 40:1 to 1.25:1 for 4 h at 37°C. Spontaneous (spont) and maximal (max) calcein release were determined by incubating targets with cRPMI alone or with 2% Triton X-100 (Sigma-Aldrich), respectively. Following incubation, the culture supernatant was assessed for calcein-AM fluorescence (F) using a spectrophotometer (PerkinElmer Victor X3 multilabel plate reader; PerkinElmer, Waltham, MA) with excitation and emission spectra of 485 and 538 nm, respectively. The percentage specific lysis for each effector-to-target ratio was calculated with the formula (F_test_ − F_spont_)/(F_max_ − F_spont_) × 100.

### Integration copy number analysis

Integration copy number was assessed using droplet digital PCR (ddPCR) as previously described.^11^ Genomic DNA (gDNA) was extracted from cryopreserved cell pellets of 5 × 10^6^ CAR T cells after 2 weeks of culture using QIAamp DNA mini kit (QIAGEN, Hilden, Germany) as per manufacturers' instructions and quantified using a Nanodrop (Life Technologies). ddPCR reaction mixes were set up containing 1× ddPCR supermix for probes (no deoxyuridine triphosphate [dUTP]; Bio-Rad, Hercules, CA), 900 nM/250 nM RPP30 primers/probe (HEX), 900 nM/250 nM CAR primers/probe (FAM), 3 IU HindIII (New England Biolabs), and 3 ng gDNA. The CAR primer/probe was designed to cover the synthetic CD28/4-1BB conjunction in the CAR construct and synthesized commercially (Bio-Rad).

Following droplet formation with the QX200 Droplet Generator (Bio-Rad), samples were transferred to a semi-skirted twin-tex 96-well PCR plate (Eppendorf, Hamburg, Germany), sealed with the PX1 PCR Plate Sealer (Bio-Rad), and amplification performed in a C1000 Touch Thermal Cycler (Bio-Rad) using the following conditions: enzyme activation (95°C for 10 min), followed by 40 PCR cycles (94°C for 30 s; 62°C for 1 min), and enzyme deactivation (98°C for 10 min), ramp rate 2°C/s. Post-amplification analysis was performed using QX200 Droplet Reader (Bio-Rad) and QuantaSoft software (Bio-Rad).

Mean integration copy number per cell was calculated by ([CAR copies/μL]/[RPP30 copies/μL]) × (2/[percentage CAR T cells by flow cytometry]).

### Integration site analysis

Integration site analysis was conducted using MuA-mediated integration site recovery as previously described.^43^ gDNA was incubated with MuA transposase (Thermo Fisher Scientific) and commercially synthesized annealed oligonucleotides (Data S1, sequence A and B).

MuA digested gDNA was amplified by PCR with a forward primer binding to either *super-piggyBac* or *piggyBat* transposon 3′-TIR (Data S1, sequence E), with the generated PCR amplicon library consisting of oligonucleotides spanning the intersection of the 3′ transposon-specific TIR and adjacent gDNA corresponding to genomic integration site. This PCR amplicon library was indexed using Nextera XT Indexing kit (Illumina, San Diego, CA) and purified using JetSeq beads (Bioline, Memphis, TN).

Next-generation sequencing of purified PCR amplicon libraries was performed on the Illumina MiSeq v.3 platform (Australian Genome Research Facility) and mapped to chromosomal loci using previously described bioinformatic algorithms.^44^ Mapped loci were annotated using HOMER hg19 peak enrichment tool,^45^ with frequency of integrations expressed as a log fold change over expected for random insertion. Raw sequencing data have been deposited in the Sequence Read Archive (Sequence Read Archive: PRJNA807916), with all unique identified integration sites provided in Table S1.

### Mouse xenograft studies

Assessment of *in vivo* activity of *piggyBat* CAR19 was conducted in a patient-derived B-ALL xenograft murine model as previously described.^12^ All animal studies were approved by the Animal Care and Ethics Committee of the University of New South Wales, and they conformed to the Animal Research Act (Australia) and the Australia Code for the Care and Use of Animals for Scientific Purposes.

Non-obese diabetic (NOD)-severe combined immunodeficiency (SCID) IL-2Rγ^−/−^ (NSG) mice were obtained from the Jackson Laboratory (Bar Harbor, ME) and bred in the animal facility at Children's Cancer Institute. Eight- to twelve-week-old mice were infused via tail vein with 3 × 10^6^ B-ALL2 cells purified from the spleen of mice transplanted with patient bone-marrow-derived leukemia blasts. Following a 1-week engraftment period, mice were randomized (five mice per group) to receive intra-peritoneal injection of either 0.33 × 10^6^ *super-piggyBac* CAR19 or varying doses of *piggyBat* CAR19 (0.17 × 10^6^ CAR low, 0.33 × 10^6^ CAR medium, and 0.66 × 10^6^ CAR high), with control groups of untreated mice and mice treated with 0.33 × 10^6^ non-specific CARs (expressing a truncated epidermal growth factor^46^ combined with the coding sequences of CD28 transmembrane and intracellular domains with CD3ζ). All doses were given based on total CAR-expressing T cells.

B-ALL and T cells were monitored by flow cytometry in peripheral blood at weekly intervals and in the bone marrow and spleen following humane culling (either due to signs of progressive leukemia, concurrent illness, or after the experimental endpoint at 11 weeks). Single-cell suspensions of bone marrow and spleen were generated by collection in PBS at room temperature and passage through 40-μm cell strainers. Red blood cells were lysed using a standard lysis buffer consisting of ammonium chloride and EDTA. Flow cytometry was used to evaluate human and mouse CD45-, CD19-, and CD3-expressing cells. Viable cells were gated using forward- and side-scatter properties, with cell suspensions stained with the following fluorochrome-conjugated monoclonal antibodies: mouse CD45-PerCP, human CD45-APC/H7, human CD19-PE, and human CD3-APC (all from BD Biosciences). CD19^pos^ and CD3^pos^ cells were analyzed in the human CD45^pos^/mouse CD45^neg^ gate. A minimum of 1 × 10^5^ events were acquired on a FACS Canto, with data analyzed with FACSDiva Software (BD Biosciences). Mice were considered leukemia free if the proportion of human CD19^pos^ cells in peripheral blood was less than 1%.

### Statistical analysis

GraphPad Prism 8 (GraphPad, La Jolla, CA) was used for graphical representation of data. SPSS Statistics 26 (IBM, Armonk, NY) was used for statistical analysis. The significance level used was p < 0.05. Each *in vitro* experiment was conducted in triplicate at a minimum (unless otherwise specified). Repeated-measures ANOVA was performed to test for systematic within-subject differences. Where a possible association was identified, Tukey's multiple comparison test was performed, with individual variances calculated for each comparison. Data are presented as mean ± SD. For *in vivo* experiments, Kaplan-Meier event-free survival (EFS) analysis was performed for each cohort of mice and the log rank test was used to compare curves. The EFS endpoint was defined as the proportion of human CD19^pos^ leukemia cells in peripheral blood reaching 20%.

## Supplemental information

Document S1. Data S1 and Figures S1–S7

Table S1. Unique integration sites identified in CD19-specific CAR T cells generated by *piggyBat* and *super-piggyBac* transposon and transposase (n = 3 donors)

Document S2. Article plus supplemental information
