# Supplementary material for: Characterizing piggyBat—a transposase for genetic modification of T cells
Source: Mol Ther Methods Clin Dev. 2022 Mar 22;25:250–63. doi: 10.1016/j.omtm.2022.03.012 (PMC9018555; doi:10.1016/j.omtm.2022.03.012)
Supplement: Document S1. Data S1 and Figures S1–S7 [file mmc1.pdf]

OMTM, Volume *25*

Supplemental information

Characterizing piggyBat—a transposase for genetic modification of T cells

Gaurav Sutrave, Ning Xu, Tiffany C.Y. Tang, Alla Dolnikov, Brian Gloss, David J. Gottlieb, Kenneth P. Micklethwaite, and Kavitha Gowrishankar

Supplementary Data / Figures

Appendix A - Oligonucleotide sequences used for integration site analysis

Sequence A -

GTAATACGACTCACTATAGGGCTCCGCTTAAGGGACTGTTTTCGCATTTATCGTG

AAACGCTTTCGCGTTTTTCGTGCGCCGCTTCA

Sequence B -

TCGGATGAAGCGGCGCACGAAAAACGCGAAAGCGTTTCACGATAAATGCGAAA

ACA[3ddC]

[3ddC] used as chain terminator

Sequence C –

GTCTCGTGGGCTCGGAGATGTGTATAAGAGACAGACGCAGACTATCTTTCTAGG

G

Sequence D -

GTCTCGTGGGCTCGGAGATGTGTATAAGAGACAGTTTGGCGGGAAATTCACC

Sequence E - TCGTCGGCAGCGTCAGATGTGTATAAGAGACAG

GTAATACGACTCACTATAGGGC

Supplementary Table 1: Unique integration sites identified in CD19 specific CAR T cells generated by *piggyBat* and *super-piggyBac* transposon/transposase (n=3 donors). Refer to supplementary file “PiggyBat and SuperpiggyBac Integration Sites.xlsx”.

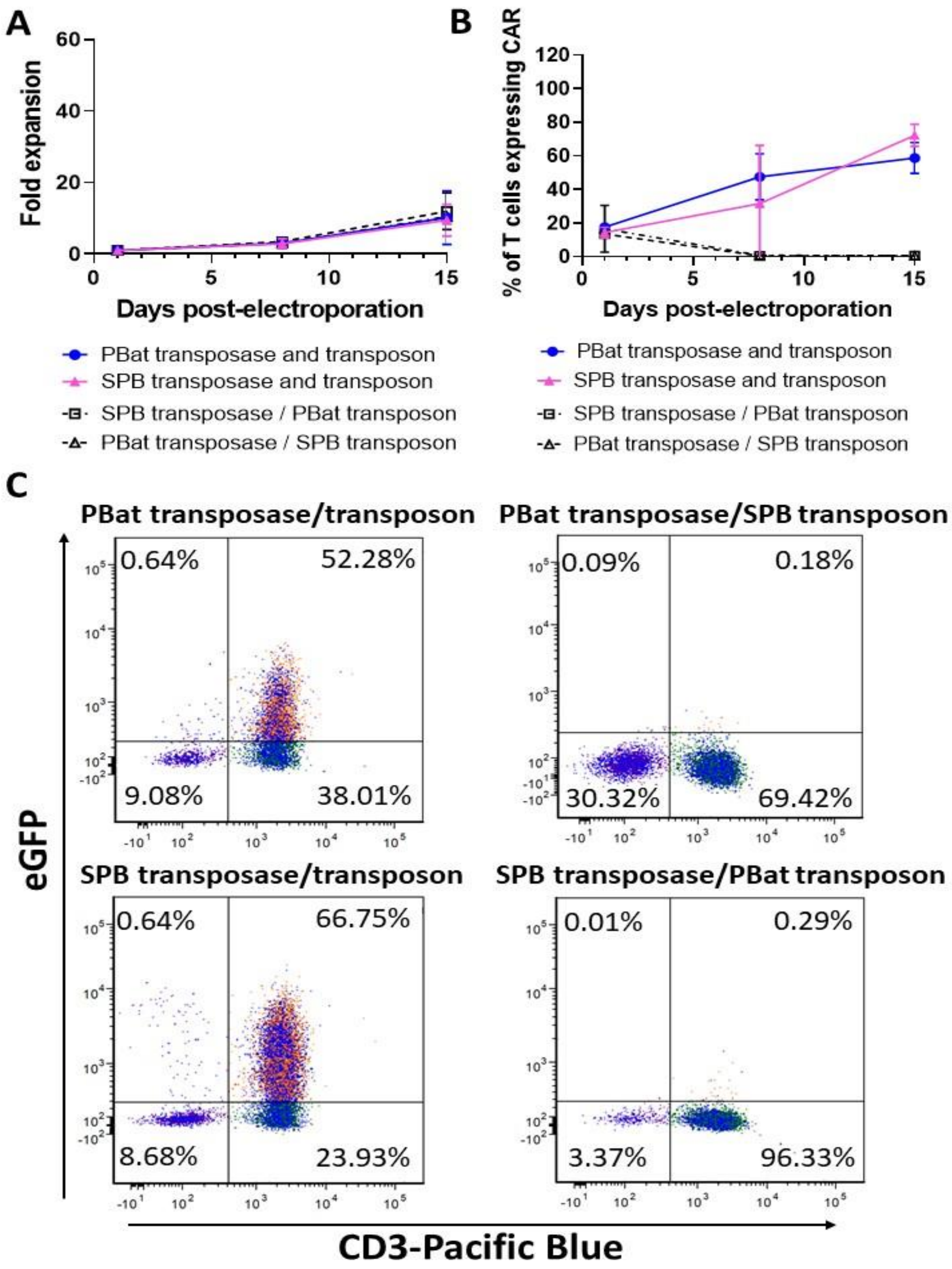

Supplementary Figure 1: PiggyBat and super-piggyBac transposase do not demonstrate cross reactivity in recognition of terminal inverted repeats (n=2 per condition). (A) Expansion and (B) CAR expression over two weeks culture following electroporation with

different combinations of transposase and transposon plasmids. (C) Representative flow plots of CAR19h28TM41BBζ-T2A-eGFP T cells at end of two weeks showing lack of eGFP expression in T cells electroporated with discordant transposase/transposon plasmids. Data presented as mean +/- standard deviation

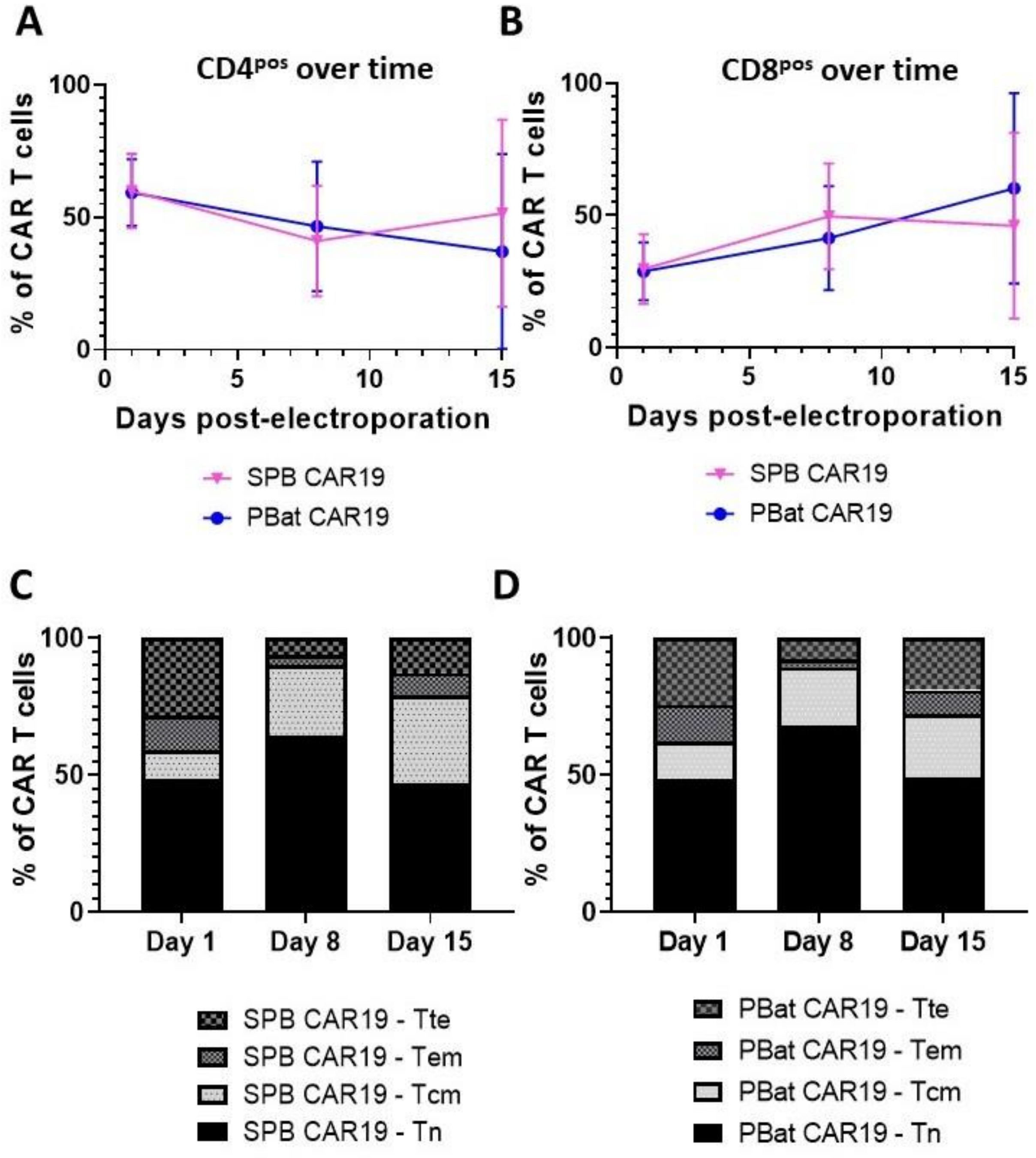

Supplementary Figure 2: Phenotypic characteristics of CAR19 T cell cultures over time. Assessed by flow cytometry of CAR19 T cells (n=3 per condition). $CD4^{pos}$ (A) and $CD8^{pos}$ (B)

and T cell immunophenotype of SPB CAR19 (C) and PBat CAR19 (D) cultures measured at weekly intervals. Data presented as mean +/- standard deviation. Tn defined as CD45RA+/CD62L+, Tcm defined as CD45RA-/CD62L+, Tem defined as CD45RA-/CD62L-, Tte defined as CD45RA+/CD62L-.

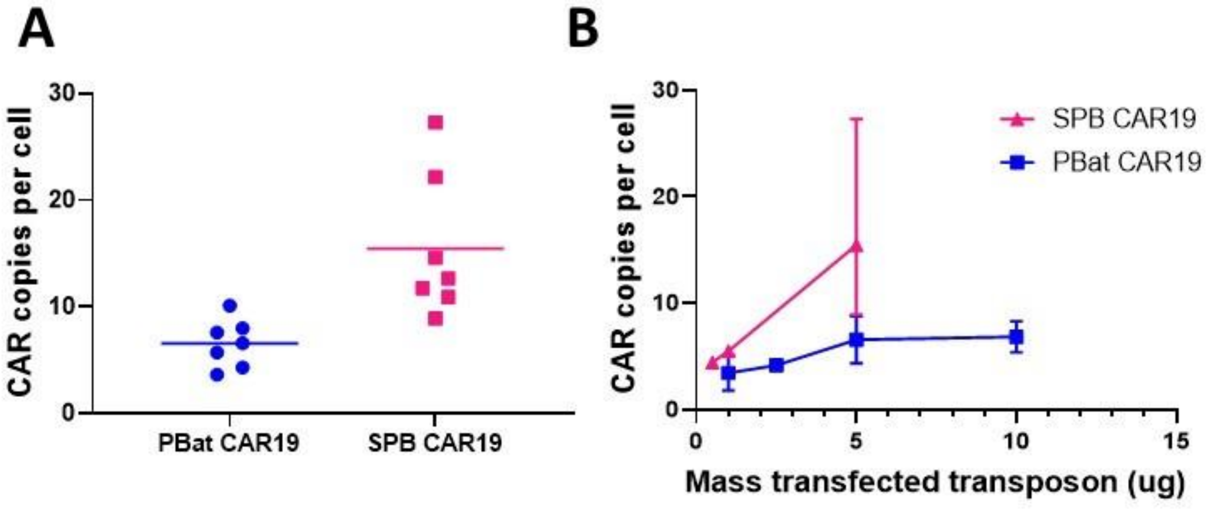

Supplementary Figure 3: CAR integration copy number varies in different donors and correlates with mass of transfected transposon plasmid. (A) CAR integration copy number in different donors. Each point represents different donor with mean (n=7). (B) CAR integration copy number increases with larger quantities of transfected transposon plasmid. Data expressed as mean +/- range. All transfections conducted with a fixed mass of 5µg transposase plasmid. SPB CAR19 demonstrate greater variability in CAR integration copy number at higher masses of transfected transposon plasmid relative to PBat CAR19.

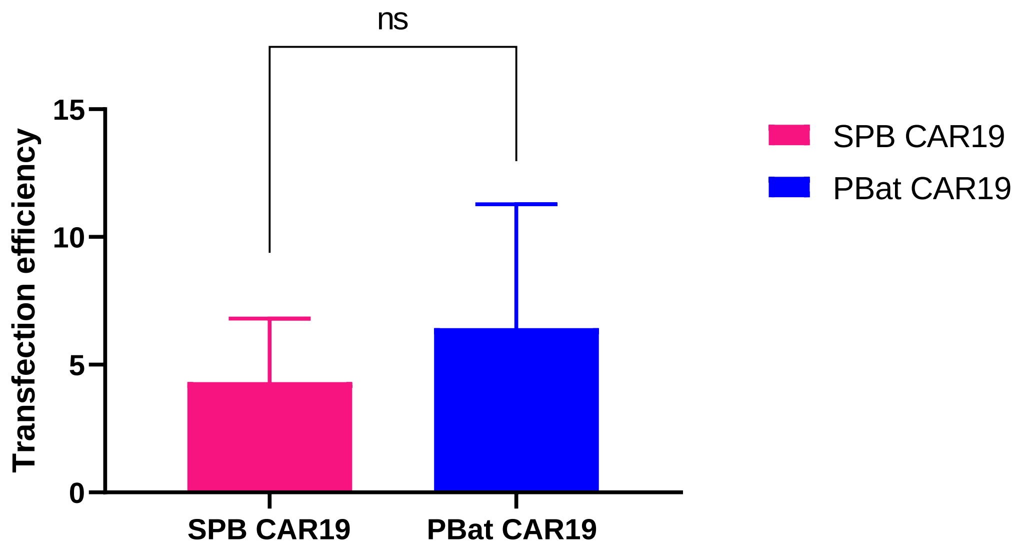

Supplementary Figure 4: Transfection efficiency for *super-piggyBac* and *piggyBat* transposase in CAR19 T cell generation (n=7). Data expressed as percentage of transfected T cells expressing CAR 24 hours post-electroporation. Mean transfection efficiency SPB CAR19 was not significantly different to that of PBat CAR19 (4.3 vs 6.4%, p=0.324)

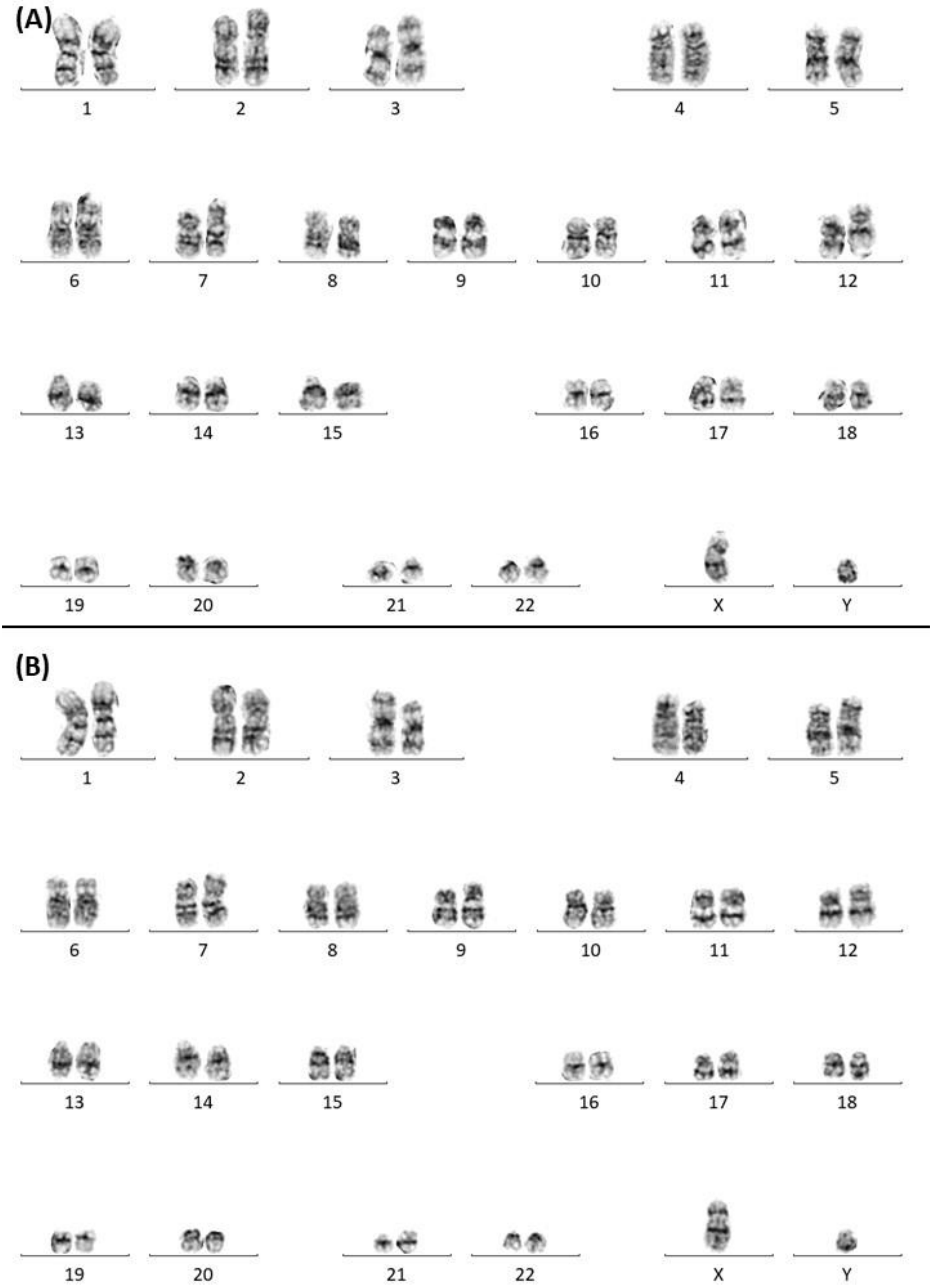

Supplementary Figure 5: Representative karyograms for *piggyBat* (A) and *super-piggyBac* (B) generated CD19 specific CAR T cells from same donor PBMCs.

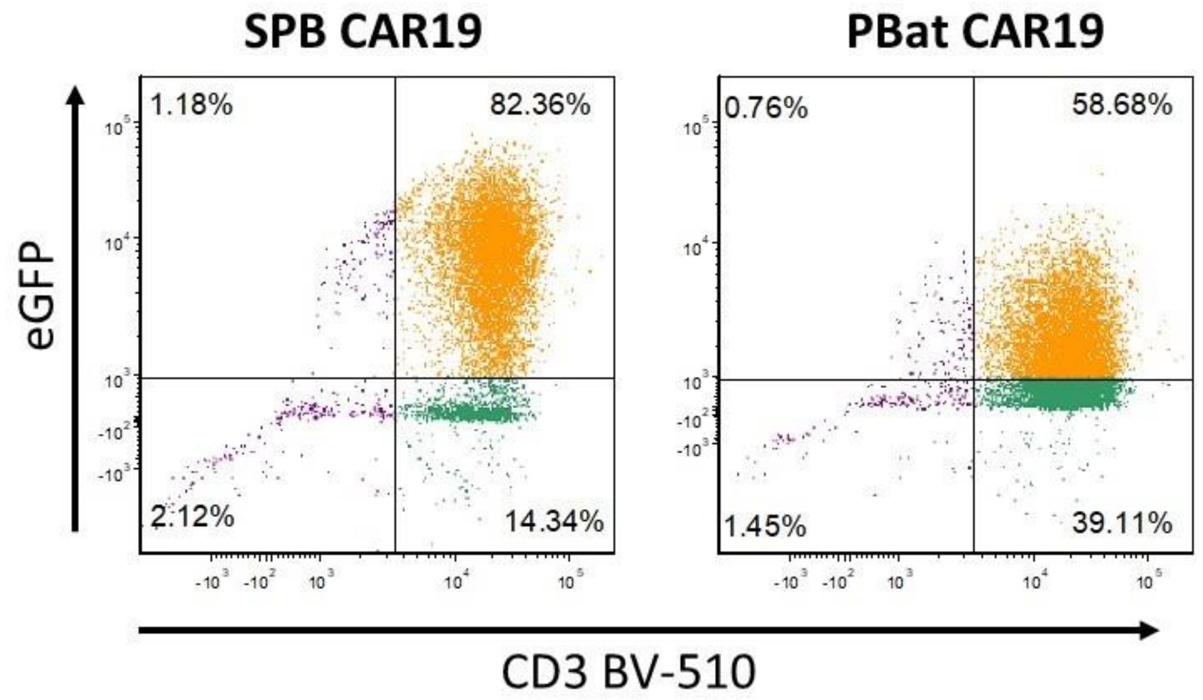

	eGFP MFI
SPB CAR19	6927.6
PBat CAR19	2006.91

Supplementary Figure 6: Representative flow cytometry plots for CAR19 T cells utilised for murine experiments.

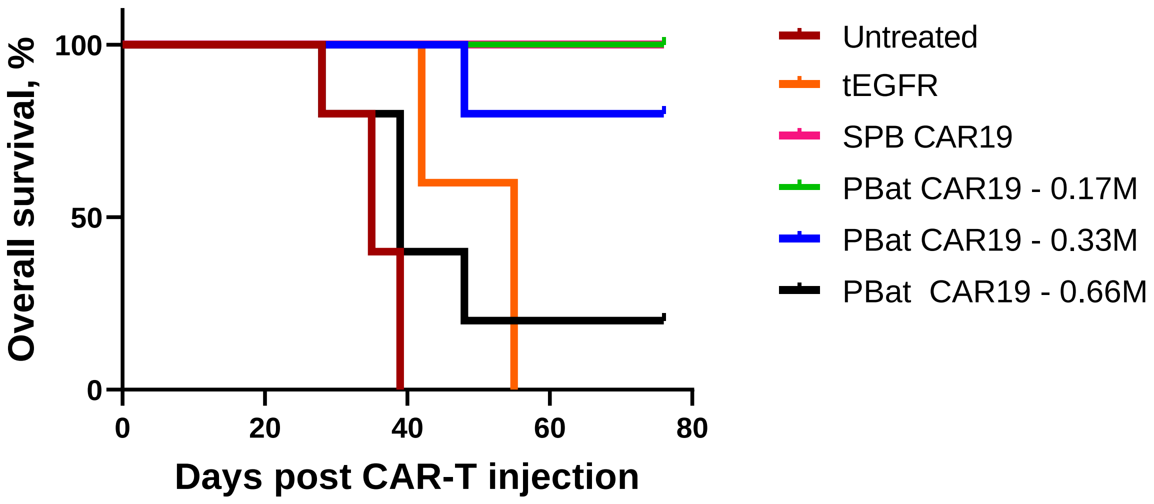

Supplementary Figure 7: Kaplan-Meier curves for overall survival following treatment (n=5 per group). SPB CAR19 and PBat CAR19 – 0.17M curves overlap
